# Mapping genetic determinants of the cell-culture growth phenotype of enterovirus 71

**DOI:** 10.1099/vir.0.029371-0

**Published:** 2011-06

**Authors:** Patchara Phuektes, Beng Hooi Chua, Sharon Sanders, Emily J. Bek, Chee Choy Kok, Peter C. McMinn

**Affiliations:** 1 Discipline of Infectious Diseases and Immunology, The University of Sydney, Sydney, Australia; 2 School of Biomedical, Biomolecular and Chemical Sciences, The University of Western Australia, Perth, Australia

## Abstract

Enterovirus 71 (EV71) is a member of the species *Human enterovirus A* within the family *Picornaviridae* and is a major causative agent of epidemics of hand, foot and mouth disease associated with severe neurological disease. Three EV71 genogroups, designated A, B and C, have been identified, with 75–84 % nucleotide sequence similarity between them. Two strains, EV71-26M (genogroup B) and EV71-6F (genogroup C), were found to have distinct cell-culture growth (26M, rapid; 6F, slow) and plaque-formation (26M, large; 6F, small) phenotypes. To identify the genome regions responsible for the growth phenotypes of the two strains, a series of chimeric viruses was constructed by exchanging the 5′ untranslated region (UTR), P1 structural protein or P2/P3 non-structural protein gene regions plus the 3′UTR using infectious cDNA clones of both virus strains. Analysis of reciprocal virus chimeras revealed that the 5′UTRs of both strains were compatible, but not responsible for the observed phenotypes. Introduction of the EV71-6F P1 region into the EV71-26M clone resulted in a small-plaque and slow-growth phenotype similar to that of EV71-6F, whereas the reciprocal chimera displayed intermediate-growth and intermediate-sized plaque phenotypes. Introduction of the EV71-26M P2–P3–3′UTR regions into the EV71-6F clone resulted in a large-plaque and rapid-growth phenotype identical to that of strain EV71-26M, whereas the reciprocal chimera retained the background strain large-plaque phenotype. These results indicate that, although both the P1 and P2–P3–3′UTR genome regions influence the EV71 growth phenotype in cell culture, phenotype expression is dependent on specific genome-segment combinations and is not reciprocal.

## Introduction

Enterovirus 71 (EV71) is a genetically diverse virus with an estimated genome evolution rate of 4.2–4.5×10^−3^ substitutions per site year^−1^ in the VP1 gene (Tee *et al.*, 2010). Three distinct EV71 genogroups, designated A, B and C, were identified by Brown *et al.* (1999). The prototype strain BrCr is the sole member of genogroup A. All other EV71 isolates belong to either genogroup B or genogroup C, which are further divided into subgenogroups B1–B5 and C1–C5, respectively (Brown *et al.*, 1999; Cardosa *et al.*, 2003; McMinn *et al.*, 2001; Shimizu *et al.*, 2004; Tu *et al.*, 2007). Co-circulation of these two distinct genogroups, B and C, in the same region has been well documented (Herrero *et al.*, 2003; Lin *et al.*, 2006; McMinn *et al.*, 2001). Viruses belonging to both genogroups were identified during the large hand, foot and mouth disease (HFMD) epidemic in Taiwan in 1998 and in several outbreaks in Malaysia from 1997 to 2000 and in Perth, Western Australia, in 1999 (Herrero *et al.*, 2003; Lin *et al.*, 2006; McMinn *et al.*, 2001). The co-circulation of both genogroups may present an opportunity for recombination events to occur.

Recombination has been reported to contribute to genetic diversity and evolution of enteroviruses (Santti *et al.*, 1999; Simmonds & Welch, 2006). Phylogenetic and SimPlot analysis of complete genome sequences of human enterovirus A (HEV-A) prototype viruses and also of the four most recently identified HEV-A viruses has suggested that recombination may play a role in the evolution of viruses within the species (Oberste *et al.*, 2004b, 2005). The occurrence of intertypic recombinants between EV71 and several HEV-A viruses, including coxsackievirus A16 (CVA16), has been demonstrated (Chan & AbuBakar, 2006; Yip *et al.*, 2010). In addition, intratypic recombination between genogroups B and C has also been identified among naturally circulating EV71 isolates in Taiwan, and two recombination sites located at the 3′ termini of proteins 2A and 3D have been identified (Huang *et al.*, 2008). The non-structural protein gene region was found to be a recombination hot spot for both inter- and intratypic recombination in EV71 and also in other HEV-A viruses. Thus, recombination events may play an important role in the emergence of EV71 subgenogroups with different virulence potential and disease associations.

During the 1999 HFMD epidemic in Western Australia, EV71 viruses belonging to genogroups B3 and C2 were isolated, indicating the co-circulation of both genogroups. Genogroup C2 viruses were isolated mainly from cases of severe neurological disease, whereas viruses belonging to genogroup B3 were isolated mainly from cases of uncomplicated HFMD and aseptic meningitis (McMinn *et al.*, 2001). Interestingly, we have found that two EV71 strains isolated during the Western Australian epidemic, 26M/AUS/2/99 (genogroup B3) and 6F/AUS/4/99 (genogroup C2), have distinct cell-culture growth phenotypes. This study aims to investigate the evolutionary relationship between these two strains and to identify genome regions responsible for the different growth phenotypes of the two strains. Chimeric recombinant viruses carrying reciprocal exchanges of the EV71-6F and EV71-26M 5′ untranslated region (UTR), the P1 structural protein gene region or the P2/P3 non-structural protein gene region plus the 3′UTR were generated, and biological properties of the parental and chimeric viruses in tissue culture were compared.

## Results

### Evolutionary and genetic relationships of EV71-6F and EV71-26M

**Fig. 1.**
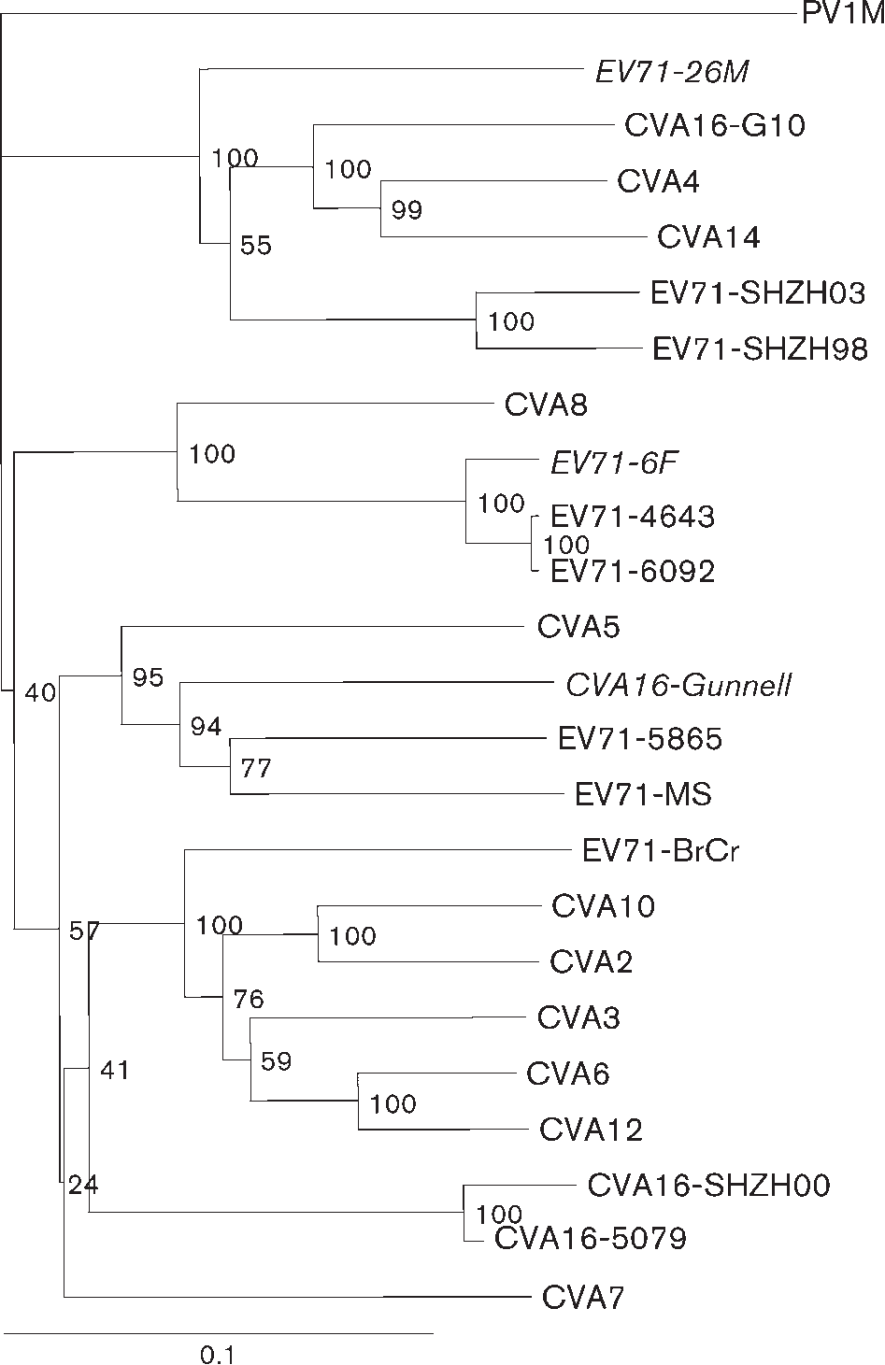
Dendrogram showing the phylogenetic relationships among 24 HEV-A viruses based on alignment of complete 3D gene sequences. The tree was constructed by neighbour joining using the Kimura two-parameter distance method (Kimura, 1980) with PV1M as the outgroup. Bootstrap values (percentages of 1000 pseudoreplicate datasets) supporting each cluster are shown at the nodes. Virus strains in italics are clinical isolates from the 1999 HFMD outbreak in Perth, Western Australia.

Based on the complete VP1 nucleotide sequence, EV71-6F and EV71-26M belong to genogroups C and B, respectively (McMinn *et al.*, 2001). However, a phylogenetic tree based on the 3D gene sequences of the same strains showed that EV71-26M (genogroup B) clustered with EV71-SHZH98, EV71-SHZH03 (both genogroup C), CVA4, CVA14 and CVA16-G10 (Fig. 1). EV71-6F and the rest of the EV71 genogroup C strains (EV71-6092 and EV71-4643) were found to cluster with CVA8; the remaining EV71 genogroup B strains were found to cluster with CVA5 and CVA16-Gunnell (Fig. 1).

**Fig. 2.**
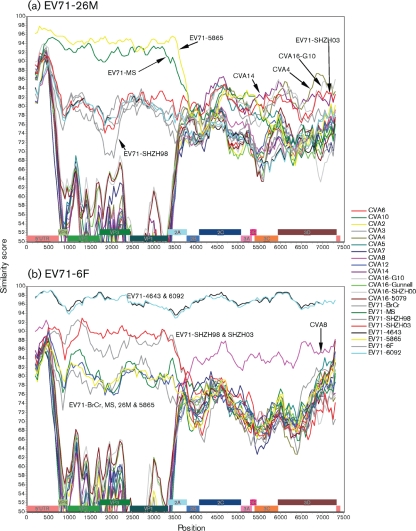
Similarity plots of HEV-A virus nucleotide sequences calculated by SimPlot 3.5.1 (Lole *et al.*, 1999). Each point represents the similarity between the query sequence [(a) EV71-26M; (b) EV71-6F] and a given heterologous sequence, within a sliding window of 400 nt centred on the position plotted, with a step of 50 residues between points. Positions containing gaps were excluded from the analysis.

These data suggest that recombination may have occurred between EV71-6F and EV71-26M and other members of the HEV-A species. Consequently, similarity plots were generated to verify this observation. Fig. 2 illustrates the genomic sequence identity of EV71-26M and EV71-6F to other HEV-A species members. The genogroup assignment of the EV71 strains based on the VP1 nucleotide sequence (McMinn *et al.*, 2001; Yan *et al.*, 2001) was also observed in the similarity plots of the P1 region, but was not preserved in the non-structural protein coding region. Furthermore, the alternative grouping of the EV71 strains based on the 3D gene sequence (Fig. 1) was also reflected in the similarity plots (Fig. 2).

Nucleotide comparison of the P2 and P3 regions revealed that EV71-26M had a higher similarity (77–88 %) to CVA4, CVA14, CVA16-G10, EV71-SHZH98 and EV71-SHZH03 than to all of the other viruses (Fig. 2a). Clustering of the EV71 genogroup C strains (EV71-6F, EV71-2086, EV71-4643 and EV71-6092) indicated a shared common ancestor between these four viruses. Interestingly, CVA8 was also identified within this grouping (Fig. 2b), suggesting that a previous recombination event may have occurred between CVA8 and an ancestor of EV71 genogroup C. These data suggest that the P2 and P3 regions of EV71-26M and EV71-6F were acquired from different ancestors through recombination, whilst the P1 region evolved through mutational drift rather than by recombination. It is possible that recombination between EV71-26M and EV71-6F and other members of the HEV-A species may have contributed to the observed differences in the growth phenotypes between these two strains.

### Comparison of growth characteristics of EV71-6F and EV71-26M

**Fig. 3.**
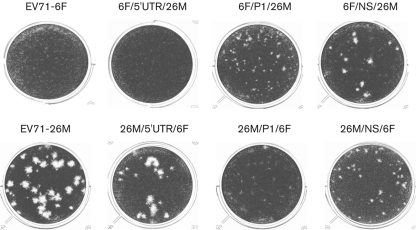
Plaque phenotype of parental EV71-6F and EV71-26M and virus chimeras on Vero cells. Tenfold serial dilutions of virus were inoculated at a volume of 100 µl per well into 12-well tissue-culture trays. Infected cells were incubated for 7 days before staining with 0.05 % (w/v) crystal violet solution. See also Table 1.

**Fig. 4.**
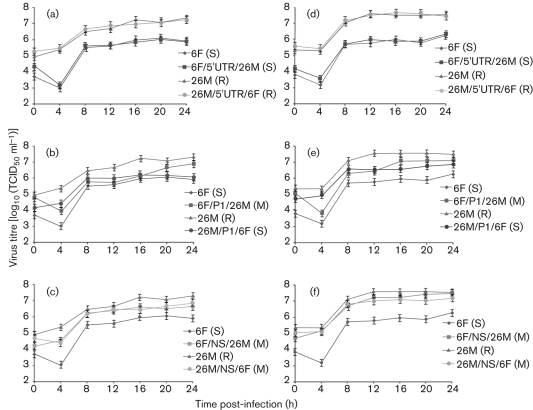
Single-step growth kinetics of parental and chimeric viruses on Vero (a–c) and RD (d–f) cells. (a, d) Growth kinetics of wild-type and 5′UTR chimera viruses. (b, e) Growth kinetics of wild-type and P1 region chimera viruses. (c, f) Growth kinetics of wild-type and non-structural protein (P2 and P3 regions) chimera viruses. Cell monolayers were infected at an m.o.i. of 5. Cell-culture supernatants were collected at the times indicated and titrated by TCID_50_ assay. All assays were performed in triplicate. At each time point, titres are means of three samples; error bars represent sem. The growth phenotypes of each chimeric virus are shown in parentheses (S, slow; M, medium; R, rapid).

Both EV71-6F and EV71-26M are able to grow on human (RD) and monkey (Vero) cells. However, during propagation of these viruses, we observed that EV71-6F grew to a maximal titre of approximately 10^6^ TCID_50_ ml^−1^ in both cell lines, whereas EV71-26M grew to higher titres of ≥10^7^ TCID_50_ ml^−1^. We investigated the phenotypic differences of these two strains by examining plaque formation and growth kinetics on RD and Vero cells. Differences in the plaque phenotype of the viruses were observed after 7 days incubation: EV71-6F displayed a pinpoint-plaque phenotype, whilst EV71-26M exhibited a large-plaque phenotype (Fig. 3; Table 1). Although the single-step growth kinetics of EV71-6F and EV71-26M are similar (Fig. 4), EV71-6F consistently grew to a final titre >10-fold lower than that of EV71-26M (*P*<0.05).

**Table 1.** Plaque phenotype of parental EV71-6F and EV71-26M and virus chimeras on Vero cells Tenfold serial dilutions of virus were inoculated at a volume of 100 µl per well into 12-well tissue-culture trays. Infected cells were incubated for 7 days before staining with 0.05 % (w/v) crystal violet solution. See also Fig. 3.

Parental backbone virus	Virus	Mean diameter of plaques (mm)^#^	Plaque phenotype
EV71-6F	EV71-6F	<0.5	Small
	6F/5′UTR/26M	<0.5	Small
	6F/P1/26M	0.9 (0.5)*	Medium
	6F/NS/26M	1.4 (0.46)	Medium
EV71-26M	EV71-26M	2.29 (0.78)	Large
	26M/5′UTR/6F	2.09 (0.76)	Large
	26M/P1/6F	<0.5	Small
	26M/NS/6F	1 (0.47)	Medium

*No. in brackets is SD.

### Generation and recovery of chimeric recombinant viruses

**Fig. 5.**
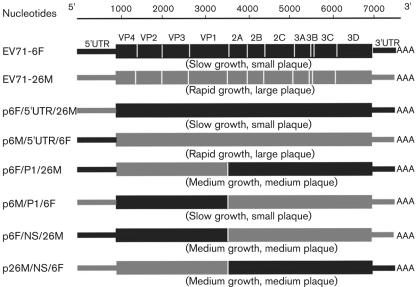
Schematic diagram of the intratypic chimera recombinant constructs between EV71-6F and EV71-26M. Chimeras are identified by using the following nomenclature: pA/B/C, where A is the backbone virus, B is the exchanged genomic region and C is the name of the virus providing the exchanged genomic region. The growth phenotypes of each chimeric virus are shown in parentheses.

In order to identify the genome regions responsible for the different growth phenotypes of EV71-6F and EV71-26M, six reciprocal chimeric recombinant infectious cDNA constructs (p6F/5′UTR/26M, p26M/5′UTR/6F, p6F/P1/26M, p26M/P1/6F, p6F/NS/26M and p26M/NS/6F) were generated by exchanging the corresponding 5′UTRs, P1 regions or P2–P3–3′UTR regions between these two strains, resulting in three reciprocal pairs of chimeric virus genomes (Fig. 5). Viable virus populations were recovered from all of the chimeric recombinant cDNA constructs. Recovered viruses were passaged five times in RD cells to increase the titre before use in subsequent assays. The plaque phenotypes of the chimeric viruses at RD cell passage 2 were found to be identical to the plaque phenotypes at the fifth passage (data not shown). In order to verify that the recovered viruses contained the expected genetic composition, RNA extracted from each recovered virus population at RD cell passage 5 was sequenced. Analysis of the sequence data obtained from the junction sites of each virus revealed that all of the recovered viruses had the expected nucleotide sequences (data not shown), and thus these virus chimera populations were used in the additional experiments described below.

### Growth characterization of chimeric recombinant viruses *in vitro*

In order to map the genetic determinants of the cell-culture growth phenotype and plaque morphology of EV71-6F and EV71-26M, the growth characteristics of the parental infectious cDNA clone-derived viruses, CDV-6F and CDV-26M, and all chimeric recombinant viruses were compared as described below. The cell-culture growth and plaque-morphology phenotypes of the wild-type and clone-derived viruses were identical (data not shown).

Mean plaque size of the parental and recombinant viruses was determined by sampling between nine and 13 plaques and the results are presented in Fig. 3 and Table 1. Parental viruses and reciprocal chimeras with a replacement in the 5′UTR displayed similar plaque morphology: CDV-6F and CDV-6F/5′UTR/26M had a pinpoint-plaque phenotype (<0.5 mm diameter), whereas CDV-26M and CDV-26M/5′UTR/6F had a large-plaque phenotype (mean size >2 mm diameter). Interestingly, the phenotype changes associated with swapping of the P1 and P2–P3–3′UTR gene regions of CDV-6F and CDV-26M were found to be non-reciprocal. The EV71-26M chimera incorporating the EV71-6F P1 region, CDV-26M/P1/6F, had a small-plaque phenotype identical to that of EV71-6F (<0.5 mm). However, the EV71-6F chimera incorporating the EV71-26M P1 region, CDV-6F/P1/26M, also displayed a small-plaque phenotype, with a mean size of 0.9±0.5 mm. The EV71-6F chimera incorporating the EV71-26M P2–P3–3′UTR regions, CDV-6F/NS/26M, exhibited a large-plaque (1.4±0.46 mm) phenotype, although this was smaller than that of EV71-26M (2.3±0.78 mm). Finally, the EV71-26M chimera incorporating the EV71-6F P2–P3–3′UTR regions, CDV-26M/NS/6F, displayed a plaque phenotype (1.0±0.47 mm) larger than that of EV71-6F (<0.5 mm).

The single-step growth characteristics of the parental and chimeric clone-derived viruses were compared in Vero and RD cells (m.o.i. of 5) and the results are shown in Fig. 4. All viruses exhibited similar growth kinetics, with titres rising from 4 h post-infection and peaking between 10^6^ and 10^7.7^ TCID_50_ ml^−1^ at 12–16 h post-infection. The reciprocal 5′UTR chimeras, CDV-6F/5′UTR/26M and CDV-26M/5′UTR/6F, displayed a growth phenotype similar to their respective parental viruses (*P*>0.05): CDV-6F and CDV-6F/5′UTR/26M produced peak titres of 10^6^ TCID_50_ ml^−1^ at 24 h post-infection in both Vero (Fig. 4a) and RD (Fig. 4d) cells, whereas CDV-26M and CDV-26M/5′UTR/6F replicated to a higher titre, with peak titres of 10^7^ and 10^7.7^ TCID_50_ ml^−1^ in Vero (Fig. 4a) and RD (Fig. 4d) cells at 24 h post-infection, respectively.

The reciprocal P1 region chimeras displayed growth characteristics intermediate to those of their respective parental viruses. The CDV-6F/P1/26M chimera replicated slowly during the initial stages of infection, producing titres of 10^5.7^ and 10^6.5^ TCID_50_ ml^−1^ in Vero (Fig. 4b) and RD (Fig. 4e) cells, respectively, at 12 h post-infection, which are comparable to those observed for the parental strain CDV-6F. However, by 24 h post-infection, CDV-6F/P1/26M had grown to a peak titre of 10^6.7^ TCID_50_ ml^−1^ in Vero cells (Fig. 4b) and 10^7.1^ TCID_50_ ml^−1^ in RD cells (Fig. 4e), similar to those produced by the other parental strain CDV-26M. The CDV-26M/P1/6F chimera produced lower peak titres at 24 h post-infection than CDV-6F/P1/26M in both Vero (Fig. 4b) and RD (Fig. 4e) cells, and these were similar to those produced by CDV-6F at the same time point.

The reciprocal P2–P3–3′UTR chimeras CDV-6F/NS/26M and CDV-26M/NS/6F displayed growth patterns similar to the parental strain CDV-26M and significantly improved growth compared with the parental strain CDV-6F in both Vero (Fig. 4c) and RD (Fig. 4f) cells at all time points examined.

Taken together, the results of plaque-morphology and single-step growth studies indicate that the 5′UTRs of both parental strains of EV71 are compatible, but are not responsible for the observed phenotypes. In contrast, the P1 and P2–P3–3′UTR genome regions exert a strong influence on the EV71 cell-culture growth and plaque-morphology phenotype.

### Comparison of translation efficiency of the EV71-6F and EV71-26M 5′UTRs

**Fig. 6.**
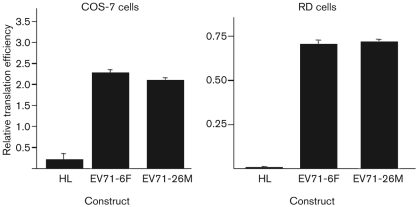
Comparative translation efficiency of the 5′UTRs of EV71-6F and EV71-26M in COS-7 and RD cells. Non-replicative bicistronic constructs HL (hairpin control), EV71-6F (containing the 5′UTR of EV71-6F) and EV71-26M (containing the 5′UTR of EV71-26M) were transfected into COS-7 and RD cells, and luciferase expression was measured by luciferase assay. Results represent means of triplicate samples; error bars represent sem.

In order to verify the compatibility of the 5′UTR between EV71-6F and EV71-26M, the efficiency of translation directed by the 5′UTRs of these two strains was compared by measuring the luciferase activity within cell lysates after the transfection of non-replicating bicistronic luciferase reporter constructs into RD and COS-7 cells. The translation efficiency of the EV71-6F 5′UTR and EV71-26M 5′UTR was found to be identical in both RD and COS-7 cells (*P*>0.05; Fig. 6). This result indicates that the ability of the 5′UTRs of these two strains to direct cap-independent [internal ribosomal entry site (IRES)-driven] translation was similar, and thus the growth defect of EV71-6F compared with EV71-26M was not likely to be determined at the level of IRES-directed translation of viral proteins.

### Comparison of kinetics of RNA synthesis of the parental and chimeric recombinant viruses

In order to examine further the genome region and underlying mechanism of phenotype expression of EV71-6F and EV71-26M, the kinetics of synthesis of positive-strand viral RNA during the first 12 h of virus replication in RD cells infected with the parental or chimeric viruses were compared by real-time RT-PCR assay.

**Fig. 7.**
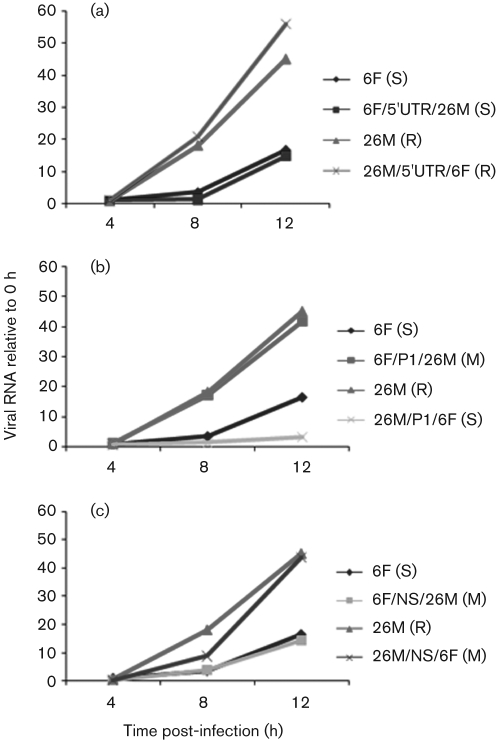
Analysis of viral positive-strand RNA synthesis during infection of RD cells. (a) RNA synthesis of parental virus and 5′UTR chimeras; (b) RNA synthesis of parental virus and P1 region chimeras; (c) RNA synthesis of parental virus and P2/P3 region chimeras. Monolayers were infected at an m.o.i. of 1. Culture supernatants were collected at the times indicated. Accumulation of positive-sense viral RNA during infection was measured by quantitative real-time RT-PCR. Yields of viral RNA at each time point were normalized to the yield at the first time point (0 h post-infection). Results represent means of duplicate samples. The growth phenotypes of each chimeric virus are shown in parentheses (S, slow; M, medium; R, rapid).

The kinetics of positive-stranded viral RNA synthesis differed significantly between CDV-6F and CDV-26M, with CDV-26M replicating much more efficiently (Fig. 7). The kinetics of positive-strand viral RNA synthesis in RD cells infected with the reciprocal 5′UTR chimeras, CDV-6F/5′UTR/26M and CDV-26M/5′UTR/6F, were indistinguishable from those observed for their respective parental viruses. Interestingly, the P1 region appeared to exert a major influence on the initiation of viral RNA synthesis in infected RD cells. RNA synthesis in CDV-26M/P1/6F-infected cells was very similar to that observed in CDV-6F-infected cells, whereas RNA synthesis in CDV-6F/P1/26M-infected cells was almost identical to that observed in CDV-26M-infected cells. In addition, the chimeras CDV-6F/NS/26M and CDV-26M/NS/6F, which retain the P1 region of their parental viruses CDV-6F and CDV-26M, respectively, displayed similar RNA-synthesis kinetics in RD cells to their respective parental viruses.

## Discussion

EV71 is a genetically diverse virus that is capable of rapid evolution (Tee *et al.*, 2010). With the exception of the prototype strain, BrCr-CA-70 (genogroup A), all known EV71 isolates belong to one of two genogroups, B and C, which have a nucleotide similarity in the range 75–84 %. We observed previously that two EV71 strains (EV71-26M and EV71-6F), isolated during the 1999 HFMD epidemic in Perth, Western Australia, displayed disparate cell-culture replication kinetics during virus propagation. These two isolates belong to two distinct genetic lineages, B3 and C2, that co-circulated during the Perth epidemic. Although EV71-26M and EV71-6F were isolated from patients with HFMD and brainstem encephalitis, respectively, the correlation of growth phenotypes and pathogenicity of EV71 strains was not confirmed (McMinn *et al.*, 2001). It was also found that some genotype B3 strains isolated in the 1999 HFMD epidemic were associated with cases of neurological disease (McMinn *et al.*, 2001). However, to date, there have been no reports describing any significant phenotypic differences associated with EV71 strains belonging to different genogroups.

Chan & AbuBakar (2006) reported that recombination had occurred between two EV71 genogroup B isolates and a close relative of the prototype CVA16 strain (CVA16-G10). A study by [Bibr r21][Bibr r22]) also reported on recombination between HEV-A species viruses. The authors showed that intratypic recombination between HEV-A species viruses was not as frequent as that seen in HEV-B. The authors suggested that this may have been due to a smaller number of individual serotypes in the HEV-A species or to the lack of temporal and geographical heterogeneity in the HEV-A species compared with the HEV-B species (Oberste *et al.*, 2004a, b). The strains EV71-6F and EV71-26M co-circulated during an HFMD epidemic in Perth, Western Australia, in 1999. Phylogenetic analyses of the VP1 and 3D nucleotide sequences together with similarity-plot data suggested that the capsid coding regions of the two viruses had evolved by genetic drift through the accumulation of point mutations, but the non-structural protein coding regions had evolved through recombination with other HEV-A species viruses. The non-structural protein region of EV71-6F appears to have been derived by recombination with a CVA8 strain, whereas the non-structural protein region of EV71-26M appears to have been derived by a complex series of recombinations involving strains CVA16, CVA4 and CVA14. These findings are consistent with earlier studies of EV71 recombination, in which intra- and intertypic recombination was observed within the P2 and P3 gene regions (Huang *et al.*, 2008; Yip *et al.*, 2010). Accumulation of mutations in the P1 region, together with intertypic and/or intratypic recombinations within the P2 and P3 regions, may allow the emergence of novel EV71 strains in the field. For example, in this study we have shown that genetically distinct P1 regions and recombinant P2/P3 regions were each responsible for the generation of EV71 field isolates with unique cell-culture growth phenotypes.

The EV71 5′UTR is known to possess critical control functions in viral protein and RNA synthesis (Andino *et al.*, 1993; Hambidge & Sarnow, 1992; Lee *et al.*, 2005; Rohll *et al.*, 1994). It contains a cloverleaf structure followed by an IRES element. The 5′ cloverleaf plays an essential role in the initiation of negative-strand RNA synthesis (Andino *et al.*, 1990; Parsley *et al.*, 1997), whilst the IRES element has a role in the initiation of translation (Ehrenfeld & Teterina, 2002). Also, the 5′UTRs of EV71-6F and EV71-26M differ significantly in length and in nucleotide sequence (85 % identity), and may be expected to confer differing growth phenotypes upon their respective virus populations. However, reciprocal 5′UTR chimeras displayed cell-culture growth and viral RNA-synthesis kinetics identical to the background virus, indicating that the 5′UTR is not involved in determining the growth phenotype of EV71-26M and EV71-6F. This could be due to the similarity of the secondary structure of the 5′UTRs of both strains (see Supplementary Fig. S1, available in JGV Online). Furthermore, the efficiency of cap-independent translation directed by the 5′UTR IRES of both viruses, when cloned into bicistronic luciferase reporter constructs, was found to be identical in transfected COS-7 and RD cells. These data indicate that the observed differences in growth phenotype of the two parental EV71 strains are unlikely to be due to differences in the control of viral protein translation, but rather to other steps in virus replication, including virus binding and entry into cells, viral RNA synthesis or virion assembly and release.

Evaluation of the cell-culture growth properties of parental EV71-26M and EV71-6F and of the chimeric recombinant viruses indicated that the P1 and P2–P3–3′UTR genome regions, but not the 5′UTR, contributed to the growth phenotype of EV71. The growth kinetics of the P1 region chimeras were found to be intermediate between those of the two parental virus strains: CDV-6F/P1/26M displayed slightly better growth than the reciprocal chimera (CDV-26M/P1/6F) in both cell lines. In contrast, the growth kinetics of the P2–P3–3′UTR chimeras were very similar to those observed for the ‘high-growth’ EV71-26M parental strain. Our findings differ from that found for CVB4, in which both the 5′UTR and P1 gene regions contributed independently to the plaque-size phenotype (Ramsingh *et al.*, 1995). The authors suggested that the 5′UTR and P1 regions both play a role in the determination of growth phenotype at different stages in the replication cycle: the 5′UTR affects an early stage in the replication cycle, whilst the P1 region may affect the efficiency of virion assembly and/or stability. However, for swine vesicular disease virus, both the P1 and P2 regions were found to be the major genetic determinants of the growth phenotype (Kanno *et al.*, 1999). These results indicate that specific genetic determinants of the growth phenotype differ between members of the family *Picornaviridae*.

As indicated above, expression of the virus growth phenotype was dependent upon specific P1 and P2–P3–3′UTR combinations within the virus chimeras and was not reciprocal. The non-reciprocal nature of the phenotypic changes conferred by forced recombination was also reported by Jiang *et al.* (2007) in a study comparing the virus growth phenotype of PV1M/CVA20 intertypic chimeras. The CVA20 P1 region was not compatible with the PV1M background strain, resulting in severely impaired or non-viable recombinant viruses. In contrast, the PV1M P1 region was fully compatible with the CVA20 background strain, producing chimeras with the parental PV1M growth phenotype. Interestingly, Jiang *et al.* (2007) showed that defective encapsidation rather than viral RNA replication contributed to the incompatibility between the CVA20 P1 region and the PV1M background. Thus, our data and those of Jiang *et al.* (2007) emphasize the importance of the role of the compatibility of specific genome-region combinations in determining the virus phenotype.

We have shown that the disparate growth phenotypes of EV71-26M and EV71-6F are not due to differences in IRES-directed translation, suggesting a role for other steps in virus replication, such as virus attachment and entry, viral RNA synthesis or virion assembly. In order to investigate the role of viral RNA synthesis as a determinant of virus growth phenotype, we examined the kinetics of RNA synthesis by using quantitative real-time RT-PCR assays. Consistent with the observed differences in virus growth in RD cells, the synthesis of positive-strand viral RNA was found to be more efficient in EV71-26M-infected than in EV71-6F-infected RD cells. As indicated previously, swapping of the 5′UTRs had no effect on the efficiency of positive-strand RNA synthesis. Interestingly, chimeras constructed by swapping the P1 regions displayed the viral RNA-synthesis phenotype of the strain donating the P1 region. In contrast, chimeras constructed by swapping of the P2–P3–3′UTR regions retained the viral RNA synthesis phenotype of the background strain. These data indicate that the P1 region has a much stronger influence on the initiation of viral RNA synthesis than the P2–P3–3′UTR regions, in contrast to the virus growth studies, which indicated a role for both the P1 and P2–P3–3′UTR regions. For example, CDV-6F/NS/26M displayed a high-growth and intermediate plaque-size phenotype in cell culture, similar to that of the EV71-26M donor, but appeared to have the highly restricted RNA-synthesis phenotype of the EV71-6F background strain. This may be due to the role played by structural proteins in mediating virus attachment and entry into cells, which influences the timing of commencement of viral RNA synthesis. Consequently, the P1 chimeras displayed RNA-synthesis kinetics identical to those of the P1 donor virus, and intermediate growth and plaque-size phenotypes due to the moderating effect of the background P2–P3–3′UTR regions, both of which support good growth in cell culture. The same holds true for the P2–P3–3′UTR region chimeras – the RNA-synthesis phenotype (high or low) is conferred by the parental P1 region, and the intermediate growth and plaque-size phenotype by the donated P2–P3–3′UTR regions.

In conclusion, the data reported in this study indicate that the P1 region is the primary determinant of the viral RNA-synthesis phenotype of EV71-26M and EV71-6F, which may reflect the influence of virus structural proteins on the early events in the infectious cycle. However, it is also possible that the P1 region may mediate improved growth in cell culture by influencing the efficiency of virion assembly late in infection. The P2–P3–3′UTR regions of EV71-26M and EV71-6F, which have been selected by unique intertypic recombination events, exert a moderating effect on the high- or low-growth phenotype of the parental viruses. The 5′UTR exerts no significant effect on the growth phenotype of either virus. Further studies are required to identify the role of early events (virus attachment, cell penetration, uncoating) and/or late events (virion assembly, release) in the expression of the growth phenotypes of both viruses.

## Methods

### 

#### Cells and viruses.

African green monkey kidney (Vero) cells (ATCC CCL-81), human rhabdomyosarcoma (RD) cells (ATCC CCL-136) and simian virus 40-transformed African green monkey kidney (COS-7) cells (ATCC CRL-1651) were maintained in Dulbecco’s modified Eagle’s medium (MultiCel; Trace Biosciences) supplemented with 5 % bovine growth serum (MultiSer; Trace Biosciences) and 2 mM l-glutamine. EV71 strains 6F/AUS/6/99 (EV71-6F; GenBank accession no. DQ381846) and 26M/AUS/2/99 (EV71-26M; accession no. AF376101) were isolated during the 1999 HFMD outbreak in Western Australia. Both strains were plaque-purified on Vero cells. Isolated plaques were passaged on RD cells to increase the titre for use in subsequent assays.

#### RNA extraction, cDNA synthesis and nucleotide sequence analysis.

Viral RNA was extracted from the culture medium of infected cells with a QIAamp Viral RNA mini kit (Qiagen) according to the manufacturer’s instructions. cDNA synthesis and nucleotide sequence analysis were performed as described previously (Chua *et al.*, 2008). Details of oligonucleotide sequences used for nucleotide sequencing are available upon request.

#### Construction of full-length infectious cDNA clones of EV71-6F and EV71-26M, and chimeric recombinant cDNA clones between EV71-6F and EV71-26M.

The full-length cDNA clones of EV71-6F (pEV71-6F) and EV71-26M (pEV71-26M) were constructed as described previously (Chua *et al.*, 2008). To facilitate the construction of chimeric recombinant viruses and subgenomic replicons, a *Bln*I restriction site was introduced into the parental clones, pEV71-6F and pEV71-26M, at the P1/P2 junction by site-directed mutagenesis, which did not cause a change in amino acid sequence.

A diagram of the chimeric recombinant cDNA clones is presented in Fig. 5. All chimeric recombinant viruses were constructed by using fusion PCR and cloning, and plasmids pEV71-6F and pEV71-26M were used as the templates. The construction procedures of each clone are described below. The name of the recombinant constructs is presented in the form pA/B/C, where A is a backbone virus, B is the exchanged genomic region and C is the name of the virus providing the exchanged genomic region.

##### p6F/5′UTR/26M.

A fragment containing part of the 5′UTR of 26M and part of the 6F P1 region was amplified by PCR. The gel-purified PCR product was used as a reverse primer for a second-round PCR, in which primer 6F-5′T7GG was used as a forward primer to amplify the whole 26M 5′UTR from the pEV71-26M template. The fusion PCR product was digested with *Sal*I and *BbvC*I^6F961^, and the *Sal*I–*BbvC*I^6F961^ fragment was cloned into *Sal*I- and *BbvC*I^6F961^-predigested plasmid pEV71-6F in a three-fragment ligation reaction.

##### p26M/5′UTR/6F.

A fragment containing part of the 6F 5′UTR and part of the 26M P1 region was amplified by PCR. The second-round PCR to amplify the full 5′UTR of 6F from the pEV71-6F template was performed using forward primer 6F-5′T7GG, and first-round PCR product as a reverse primer. The fusion PCR product was digested with *Sal*I and *Bsiw*I^26M1442^, and a *Sal*I–*Bsiw*I^26M1442^ fragment was cloned into *Sal*I- and *Bsiw*I^26M1442^-predigested plasmid pEV71-26M.

##### p6F/P1/26M.

The fragment containing the 6F 5′UTR adjacent to the 5′ terminus of 26M P1 was amplified by PCR. This fragment was then used in the second-round PCR as a forward primer, and 6FP126MR2 (containing a *Bln*I site) was used as a reverse primer. The fusion PCR product containing the 6F 5′UTR and 26M P1 was digested with *Sal*I and *Bln*I^26M3333^, and a gel-purified *Sal*I–*Bln*I fragment was cloned into pEV71-6F to generate p6F/P1/26M.

##### p26M/P1/6F.

The fragment containing the 26M 5′UTR adjacent to the 5′ terminus of 6F P1 was amplified by PCR. This fragment was then used for a second-round PCR with a reverse primer, 26MP16FR2. The fusion PCR product containing the 26M 5′UTR and the 6F P1 was digested with *Sal*I and *Bln*I, and a gel-purified *Sal*I–*Bln*I^6F3330^ fragment was then cloned into pEV71-26M to generate p26M/P1/6F.

##### p6F/NS/26M.

p6F/NS/26M was constructed by digestion of plasmid pEV71-26M with *Bln*I and *Mlu*I. The excised *Bln*I^26M3333^–*Mlu*I fragment was then cloned into *Bln*I6F^3330^- and *Mlu*I-predigested plasmid pEV71-6F to produce p6F/NS/26M.

##### p26M/NS/26M.

p26M/NS/26M was constructed by digestion of plasmid pEV71-6F with *Bln*I and *Mlu*I. The excised *Bln*I^6F3330^–*Mlu*I fragment was then cloned into *Bln*I^26M3333^- and *Mlu*I-predigested pEV71-26M to produce p26M/NS/6F.

#### Transfection and recovery of clone-derived virus populations.

Transfection of the full-length cDNA clones was undertaken in COS-7 cells using Lipofectamine 2000 (Invitrogen) as described previously (Chua *et al.*, 2008). Clone-derived viruses were then passaged on RD cells five times to increase virus titres and stored at −80 °C until required.

#### Construction of bicistronic constructs.

A plasmid containing the *Renilla* luciferase (RLuc) and firefly luciferase (FLuc) genes was a gift from Anne-Catherine Prats, Hôpital de Rangueil, Toulouse, France. RLucR is controlled by the cytomegalovirus immediate-early promoter and FLucF is controlled by an RNA hairpin (negative control). The EV71-6F and EV71-26M bicistronic constructs were constructed by replacing an RNA hairpin structure with the 5′UTRs of EV71-6F and EV71-26M, respectively. A diagram of the bicistronic constructs is presented in Supplementary Fig. S2 (available in JGV Online).

#### Luciferase assay.

RD or COS-7 cells were seeded onto 24-well plates and incubated for 24 h to reach about 90 % confluence at the time of transfection. One microgram of each bicistronic construct and 3 µl Lipofectamine 2000 were transfected into the cells as described previously (Chua *et al.*, 2008). Cells were assayed for luciferase activity at 24 h post-transfection using dual luciferase reagents (Promega), following the manufacturer’s protocol. Luciferase activity was quantified by using a luminometer (Ascent) or microplate reader (FLUOstar).

#### Virus titration.

Virus titres were determined by measuring TCID_50_ as described previously (Chua *et al.*, 2008).

#### Single-step growth kinetics and plaque assays.

Single-step growth kinetics were determined on Vero and RD cell monolayers grown overnight in 48-well tissue-culture trays (4×10^4^ and 5×10^4^ cells per well, respectively) as described previously (Chua *et al.*, 2008). Plaque assays were performed on Vero and RD cells, as described by Arita *et al.* (2005).

#### Real-time RT-PCR for viral RNA quantification.

RD cell monolayers were infected with viruses at an m.o.i. of 1, and viral RNA was extracted from the culture medium of infected cells at various time points by using a QIAamp Viral RNA mini kit (Qiagen). Real-time RT-PCR for the detection of the 5′UTR of EV71 was performed as described by Nijhuis *et al.* (2002). The first strand of cDNA was synthesized by using Moloney murine leukemia virus reverse transcriptase for RT-PCR (Promega) and reverse primer. Real-time RT-PCR was performed in a 12.5 µl reaction mixture containing 2 µl cDNA solution and 6.5 µl QuantiTect Probe PCR master mix (Qiagen) with a forward primer (10 pmol), reverse primer (10 pmol) and probe (5 pmol). Plasmid construct pEV71-6F was used to control the quantification of the number of copies. The mixtures were subjected to real-time PCR; the PCR conditions consisted of a denaturation step at 95 °C for 10 s and 40 cycles of 95 °C for 15 s and 60 °C for 60 s. The fluorescence emission of the probe was monitored and analysed by using a Corbett Rotor-Gene 6000 (Corbett Life Science).

#### Phylogenetic analysis.

All available complete genome sequences of HEV-A species viruses, with the exception of HEV77, were obtained from GenBank (see Supplementary Table S1, available in JGV Online) and phylogenetic analysis was performed. EV71 sequences with <1 % full genome sequence divergence from each other were excluded. The 3D gene sequences were aligned by using the clustal w program (Thompson *et al.*, 1994). Phylogenetic trees were constructed by using neighbour joining with the Kimura two-parameter distance method (Kimura, 1980) and viewed using the TreeView program (Page, 1996). PV1M was included as an outgroup. Bootstrap verification of the resulting phylogenetic tree was performed by analysis of 1000 bootstrapped pseudoreplicates. Consensus trees were subsequently produced and viewed by using the TreeView program.

#### Similarity analysis.

Similarity analysis of the complete genome alignments was performed with the SimPlot software package version 3.5.1 (Lole *et al.*, 1999). Similarity was calculated in each window of 400 nt by the Kimura two-parameter distance method (Kimura, 1980) with a transition–transversion ratio of 2, and the window was advanced successively along the genome alignment in 50 nt increments.

#### Statistical analysis.

Statistical analysis was performed using Student’s *t*-test and analysis of variance (ANOVA).
